# Adipose tissue: an inflammatory organ that can not be ignored in periodontal disease related to obesity

**DOI:** 10.3389/fimmu.2025.1698207

**Published:** 2025-10-31

**Authors:** Qiqi Wang, Hongyan Li, Yu Huan, Tianyu Zhou, Jingdan Zhang, Rongkaixuan Fang, Yue Sun, Lan A, Wenzhou Xu

**Affiliations:** ^1^ Department of Periodontology, Hospital of Stomatology, Jilin University, Changchun, Jilin, China; ^2^ Jilin Provincial Key Laboratory of Tooth Development and Bone Remodeling, Hospital of Stomatology, Jilin University, Changchun, Jilin, China; ^3^ Department of Oral Implantology, Hospital of Stomatology, Jilin University, Changchun, Jilin, China

**Keywords:** adipose tissue, periodontitis, obesity, chronic inflammation, immune metabolic disorder

## Abstract

In obesity, the pathological remodeling of adipose tissue characterized by hyperplasia and hypertrophy serves as a critical hub driving chronic inflammation. This process triggers adipose microenvironment disruption, manifesting as reduced angiogenesis, excessive extracellular matrix deposition, dysregulated adipokine secretion, and enhanced immune cell infiltration, ultimately leading to a systemic low-grade inflammatory state. Functioning as an active inflammatory organ, dysfunctional adipose tissue specifically exacerbates periodontitis progression through multiple mechanisms: including glucose/lipid metabolic imbalance, dysregulated bone metabolism with imbalanced osteoclast-osteoblast activity, immunometabolic disturbances, microcirculatory impairment, degradation of periodontal extracellular matrix and dysfunction of epithelial barrier and gut microbiota dysbiosis. This review systematically elucidates the interactive mechanisms between adipose tissue-derived inflammatory signaling and periodontal pathology, emphasizing its central role in obesity-associated periodontal diseases. Based on these mechanisms, we propose targeted intervention strategies: modulating adipokine secretion, suppressing immune cell infiltration in adipose tissue or restoring adipose tissue metabolic homeostasis may emerge as novel approaches to disrupt the obesity-periodontitis vicious cycle.

Future studies might enhance the clinical translation of multi-organ treatment approaches that target the adipose tissue-periodontium axis while continuing to explore the regulatory effects of immune pathways specific to adipose tissue on the periodontal microenvironment.

## 1 Introduction

Both obesity and periodontitis are highly prevalent chronic diseases worldwide, and their correlation poses a significant public health burden. In recent years, systemic low-grade inflammation, the core pathophysiological link between the two, has gained considerable scientific interest. Studies show that prolonged innate immune activation is associated with obesity, diabetes, and smoking, highlighting the central role of chronic inflammation in disease development (1, 2). Obesity, defined as “excessive fat accumulation” (3), is a well-established risk factor for periodontitis (4–6). Its key pathological feature is the dysfunctional expansion of adipose tissue (AT), characterized by inadequate angiogenesis, excess extracellular matrix (ECM) deposition, and abnormal immune cell infiltration, which together trigger local chronic inflammation (7).

Far from being a passive energy reservoir, AT is an active endocrine-immune hub (8). Under physiological conditions, its rich resident network of diverse immune cells, such as innate and adaptive immune cells alongside lymphoid structures, helps maintain immune homeostasis, suppress excessive inflammation, and participate in tissue remodeling. However, in the obese state, hypertrophied adipocytes become dysfunctional, driving massive abnormal infiltration of immune cells, predominantly macrophages that accumulate particularly in visceral fat. This results in the sustained activation of this critical inflammatory hub. The endocrine-immune network within dysfunctional AT becomes imbalanced. This imbalance is characterized by increased secretion of pro-inflammatory adipokines alongside decreased levels of anti-inflammatory factors, such as adiponectin, and the release of large quantities of pro-inflammatory cytokines, such as Tumor Necrosis Factor-alpha (TNF-α) and Interleukin-6 (IL-6) (9).

Thus, dysfunctional AT acts as a key inflammatory hub, driving systemic low-grade inflammation. It promotes metabolic dysfunctions such as insulin resistance and affects distant organs, including periodontal tissues, via mechanisms like glucose/lipid dysregulation, bone metabolism imbalance, immune-metabolic disruption, and gut microbiota–immune axis alterations (10–14). Notably, adipose-derived inflammatory factors can disturb the osteoclast–osteoblast balance, directly influencing periodontal bone homeostasis (15). Periodontitis, a chronic inflammatory disease triggered by oral dysbiosis (16), interacts with obesity in a destructive synergy: inflammatory signals from the adipose hub amplify local periodontal inflammation, accelerating tissue breakdown (17).

In summary, dysfunctional AT plays an indispensable role in linking obesity and periodontitis. Although their epidemiological association is clear, the specific endocrine and immune mechanisms through which AT influences periodontitis remain underexplored. This review highlights clinical and experimental evidence underscoring AT’s pivotal role in the development and progression of obesity-related periodontitis. A deeper understanding of how this inflammatory hub affects periodontal health will be crucial for designing targeted prevention and treatment strategies for obese individuals.

## 2 Heterogeneity and plasticity of AT

**Figure 1:**
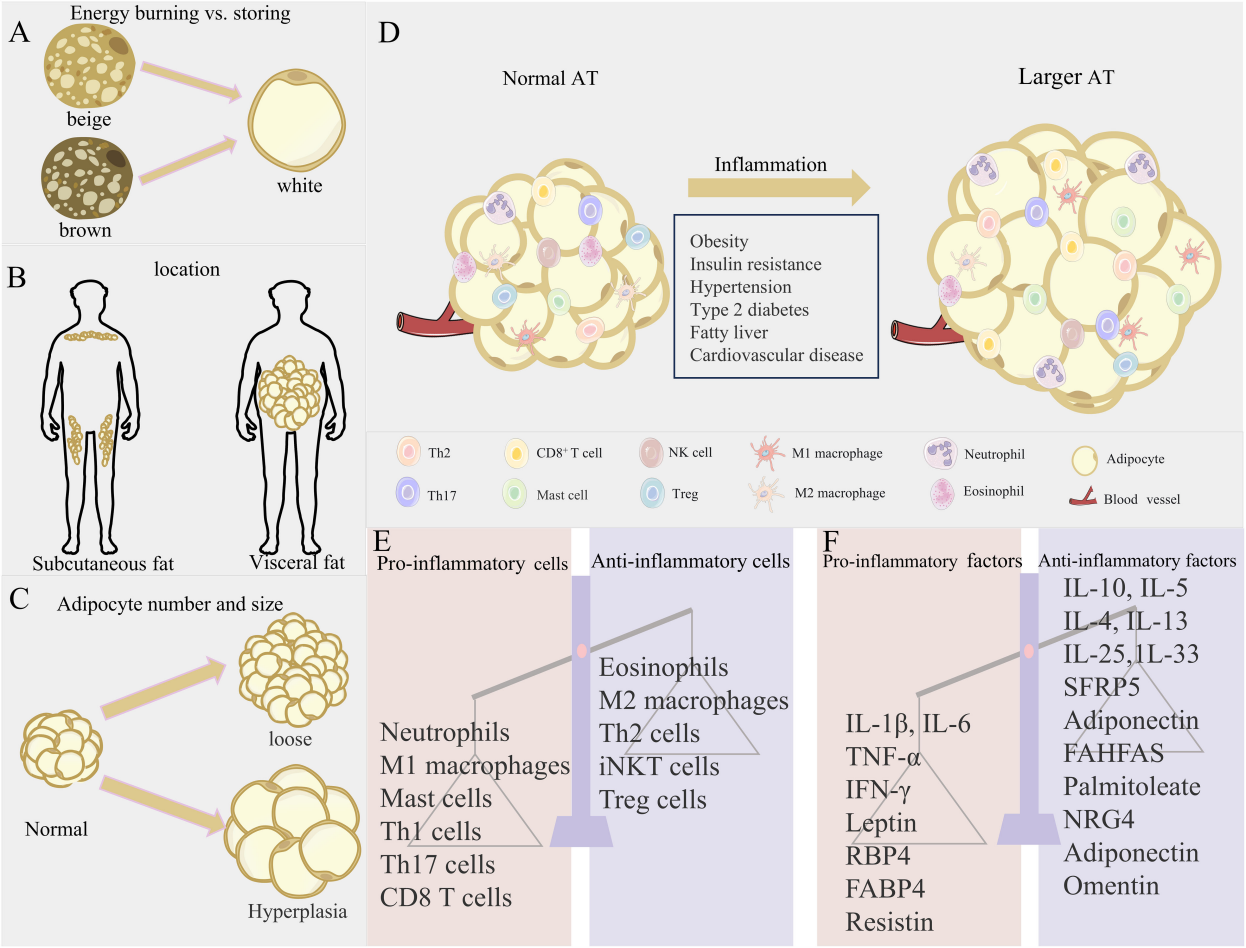
Obesity-induced changes in AT location and microenvironment. **(A–C)** Three axes of AT variation: expansion mechanism (hypertrophy vs. hyperplasia), anatomical location (visceral vs. subcutaneous), and metabolic phenotype (energy storage vs. energy burning), contrasting metabolically favorable (left) and unfavorable (right) states. **(D)** Immune cell shift from anti-inflammatory profiles in lean AT to pro-inflammatory dominance in obesity. **(E, F)** Resultant dysregulation of adipose factors and immune imbalance.

White adipose tissue (WAT), brown adipose tissue (BAT), and beige adipose tissue (BeAT) are the principal classifications of AT, each exhibiting unique morphological and functional attributes. WAT appears white and functions as the body’s principal energy reserve, providing cushioning, protection, and thermal insulation (18). Moreover, AT serves as an essential endocrine organ, producing and secreting several peptide hormones and signaling chemicals termed adipokines, which profoundly influence metabolism (especially lipid metabolism), inflammation, immunological responses, and fibrinolysis. White adipocytes, defined by their single large lipid droplet, fulfill core physiological roles by storing triglycerides and liberating fatty acids to meet energy demands. BAT appears brown due to its high density of mitochondria and blood vessels, and its core function is energy dissipation via uncoupled thermogenesis rather than energy storage (19). Brown adipocytes are multilocular, abundant in mitochondria, and generate heat through uncoupling protein-1 (UCP-1) uncoupling, demonstrating elevated glucose uptake and oxidation features. BeAT is primarily found within WAT depots. Under basal conditions, beige adipocytes exhibit a unilocular morphology. However, upon stimulation by cold or catecholamines, they can be activated, acquiring characteristics similar to BAT, such as enhanced lipolysis, increased mitochondria, UCP-1 expression, and formation of small lipid droplets, thereby gaining thermogenic capacity (largely dissipated as heat) (20). Notably, unlike brown adipocytes, which primarily rely on UCP-1, beige adipocytes also utilize alternative thermogenic pathways such as the creatine futile cycle and calcium futile cycling (**Figure 1A**) (21).

AT is widely distributed throughout the body. WAT is predominantly located in subcutaneous and visceral regions. Subcutaneous WAT accounts for over 80% of total body fat, while visceral WAT comprises approximately 10% in females and 20% in males. BAT and BeAT are mainly distributed in specific regions such as the paravertebral, cervical/supraclavicular, peritracheal/perivascular, and perirenal/periadrenal areas (**Figure 1B**). The thermogenic activity of brown and beige fats is helpful to fight obesity and related metabolic disorders, such as hyperglycemia and hyperlipidemia (20). Interscapular-deposited BAT orchestrates systemic glucose and lipid homeostasis, rendering its targeted activation a validated anti-obesity therapeutic approach. Fat distribution is crucial for metabolic risk and exhibits depot-specific effects. Central obesity, characterized by visceral fat accumulation, significantly elevates risks of insulin resistance, atherosclerosis, diabetes, and metabolic syndrome (22). In contrast, subcutaneous fat accumulation generally carries lower metabolic risk. Excessive hypertrophy or hyperplasia of WAT is closely associated with its dysfunction and is a key factor in obesity, metabolic syndrome, and type 2 diabetes. Among these, visceral adipocyte hypertrophy is more prone to inducing insulin resistance and cardiometabolic disturbances, while subcutaneous adipocyte hypertrophy is primarily associated with insulin resistance (**Figure 1C**) (23). Although BAT is less susceptible to obesity-induced inflammation than WAT, severe obesity can still induce a local pro-inflammatory environment within BAT, impairing its thermogenic capacity and glucose uptake, while inflammatory cytokines can also hinder beige fat formation (21).

AT exhibits high plasticity and remodeling capacity. Adipocytes adapt to energy fluctuations through hypertrophy (increasing cell volume) and hyperplasia (24). Under normal conditions, AT maintains energy homeostasis by absorbing or releasing nutrients (25–28). These three types of adipocytes/tissues work synergistically to maintain metabolic balance. However, sustained lipid overload leads to pathological remodeling, characterized by reduced angiogenesis, ECM deposition, increased immune cell infiltration, and chronic low-grade inflammation (7). This allostatic overload disrupts metabolic and cardiovascular regulation. When one or more adipose depots become dysfunctional, it can lead to insulin resistance and metabolic complications.

## 3 Immune and endocrine functions of AT

### 3.1 Immune functions of AT

AT functions as a dynamic immune-endocrine nexus with intrinsically interconnected roles. This organ harbors diverse innate and adaptive immune populations, including but not limited to macrophages, neutrophils, dendritic cells, and T/B lymphocytes. Under physiological conditions, these cells orchestrate tissue homeostasis through critical processes such as extracellular matrix assembly, insulin sensitivity modulation, and apoptotic cell clearance. Here, M2-polarized macrophages predominate, actively secreting anti-inflammatory mediators (29). The tissue further develops secondary lymphoid-like structures exemplified by fat-associated lymphoid clusters (FALCs), which fine-tune adaptive immunity to preserve metabolic equilibrium (30).

However, the AT “microenvironment” is relatively fragile. In obesity, the excessive expansion of WAT reaches its limit, leading to adipocyte hypertrophy, death, and fibrosis, triggering unresolved chronic low-grade inflammation (31). Central to this inflammation is skewed immunity wherein pro-inflammatory cells (neutrophils, M1 macrophages, and Th1/CD8^+^ T cells) surge while anti-inflammatory regulators (Tregs and M2 macrophages) diminish, creating pathological polarization (31). Nutrient-sensing pathways mediate the direct effects of lipid/glucose excess on critical T cell subsets (Th17, Tregs) during differentiation and functional regulation (32), while propagating macrophage-dominated adipose inflammation through M1-polarized expansion (33). This immune imbalance accelerates pro-inflammatory responses and AT dysfunction (**Figure 1D**) (31).

### 3.2 Endocrine functions of AT

Adipokines, which include TNF-α, IL-6, adiponectin, leptin, fatty acid binding protein 4 (FABP4), chemerin, fibroblast growth factor 21 (FGF21), and others, are released by AT, which is also an important endocrine organ (34–38). In obesity, adipose tissue macrophages (ATMs) become the predominant leukocytes infiltrating the tissue, their numbers positively correlating with the degree of obesity and adipocyte size (39). These polarized, pro-inflammatory ATMs are the primary source of local and circulating inflammatory cytokines, particularly TNF-α and IL-6 (40, 41). Studies show that AT from obese individuals contributes up to 50% of circulating IL-6 levels, making it a key driver of systemic inflammatory status (39).

Adipocytes and macrophages form a close interaction network within obese AT, jointly driving inflammation (33). It is believed that hypertrophic adipocytes release chemokines that attract macrophages. Once macrophages arrive, they change into the pro-inflammatory phenotype (M1) and start making inflammatory adipokines (33, 41). Key pro-inflammatory adipokines like TNF-α and IL-6 not only make local inflammation and insulin resistance worse, but they also get into the bloodstream and have big effects on both metabolic balance and the inflammation cascades (33, 42). In order for AT to grow normally, inflammatory processes need to be tightly controlled. Blocking inflammatory pathways, for example by transgenic expression of the anti-inflammatory protein RIDα−RIDβ, makes adipogenesis and homeostasis less effective (42). Adipose-derived bioactive metabolites, such as fatty acids and leukotrienes, actively regulate inflammatory cascades and metabolic alterations, thereby accelerating metabolic syndrome pathogenesis (42, 43).

In conclusion, dysregulation of the immune-endocrine network constitutes the core pathology of obesity-induced AT dysfunction. Adipocyte hypertrophy and death initiate abnormal recruitment and M1-polarized pro-inflammatory activation of immune cells, particularly macrophages, driving hypersecretion of key inflammatory adipokines such as TNF-α and IL-6. This generates both a local self-perpetuating cycle that exacerbates insulin resistance and tissue damage and significant systemic low-grade chronic inflammation (33, 40, 41). Such inflammatory states represent a critical pathological foundation for obesity-associated metabolic disorders, including insulin resistance and cardiovascular disease, alongside elevated autoimmune susceptibility (**Figures 1E, F**) (42, 44–46).

## 4 Evidence linking AT, obesity, and periodontitis

### 4.1 Epidemiological evidence

Both obesity and periodontitis represent highly prevalent chronic diseases globally. Beyond being a significant risk contributor to systemic conditions including type 2 diabetes and cardiovascular diseases, obesity demonstrates a well-established epidemiological association with periodontitis onset and progression, thereby constituting an independent risk determinant for periodontal pathology (47).

Animal studies demonstrate that obesity, especially when concurrent with hypertension, aggravates experimental periodontitis progression and induces destructive changes in periodontal tissues, notably including diminished alveolar bone mass (43). Weight gain itself is associated with reduced alveolar bone height, suggesting that obesity may negatively affect periodontal tissues even in the absence of clinical disease (43).

Numerous population-based studies have further validated and extended these findings: Elevated body mass index (BMI), waist circumference (WC), body fat percentage, and serum lipid levels are all significantly and positively associated with the risk of periodontitis (48). Obesity can elevate an individual’s risk of getting periodontitis by 2 to 3 times, independent of characteristics such as age, gender, and smoking (49). Data from NHANES III suggest that body fat content is significantly associated with periodontal disease even in younger populations (49). A well-documented positive correlation exists between key obesity indices such as BMI and waist-to-hip ratio and periodontal markers including clinical attachment loss and probing depth. Crucially, central adiposity, or elevated systemic fat burden, significantly elevates periodontitis risk, even among adults exhibiting metabolically healthy phenotypes. Echocardiographic measurement of epicardial AT thickness also appears to be associated with severe periodontitis (50).

In summary, evidence from both animal models and human studies demonstrates that obesity plays a pivotal role in increasing the risk of periodontitis through its core pathological basis-AT dysfunction. Concurrently, the oral microbiota associated with periodontitis and the chronic systemic inflammation triggered by bacterial lipopolysaccharides (LPS) entering the bloodstream may also exacerbate AT inflammation, potentially forming a vicious cycle (51).

### 4.2 Potential mechanisms by which AT influences periodontitis

#### 4.2.1 Bone metabolism imbalance

The interaction mechanisms between AT and bone metabolism can be understood through two perspectives: cell differentiation and endocrine regulation. At the cellular differentiation level, AT, as a significant source of multipotent mesenchymal stem cells (MSCs), exhibits a differentiation fate closely linked with bone metabolism. Studies indicate that MSCs within AT possess the potential to differentiate into adipocytes, osteoblasts, and chondrocytes. This multipotent differentiation capacity forms the cellular basis for their connection with bone metabolism (52). It’s important to note that the adipogenic and osteogenic differentiation of MSCs work together to keep a balance: signals for adipogenic differentiation stop osteogenic differentiation, and signals for osteogenic differentiation stop adipogenesis (53). The clinical significance of this competitive differentiation is evident in the observation that abnormal hyperplasia of AT is often accompanied by decreased bone formation capacity. Histomorphological studies confirm a significant negative correlation between fat mass and bone formation (54). During periodontal tissue regeneration, disruption of this differentiation balance may impair bone regeneration, suggesting a potential role for adipose-bone metabolic imbalance in periodontitis-induced bone defects (11).

At the molecular regulatory level, AT regulates bone metabolism via paracrine/endocrine mechanisms by secreting various adipokines. On one hand, pro-inflammatory cytokines secreted by AT (such as TNF-α and IL-6) promote osteoclast formation by activating the RANKL/RANK/OPG pathway while also interfering with STAT signaling and disrupting bone homeostasis (55–57). Animal experiments confirm that high-fat diet-induced obesity models display abnormal bone metabolism, including trabecular bone structure deterioration and impaired bone formation, observable within a short period, alongside elevated serum leptin levels (58). On the other hand, weakened anti-inflammatory mechanisms combined with increased pro-inflammatory factors lead to chronic low-grade inflammation. This pathological feature is prevalent in both obesity and periodontitis (59, 60) and synergistically exacerbates alveolar bone resorption (61).

**Figure 2:**
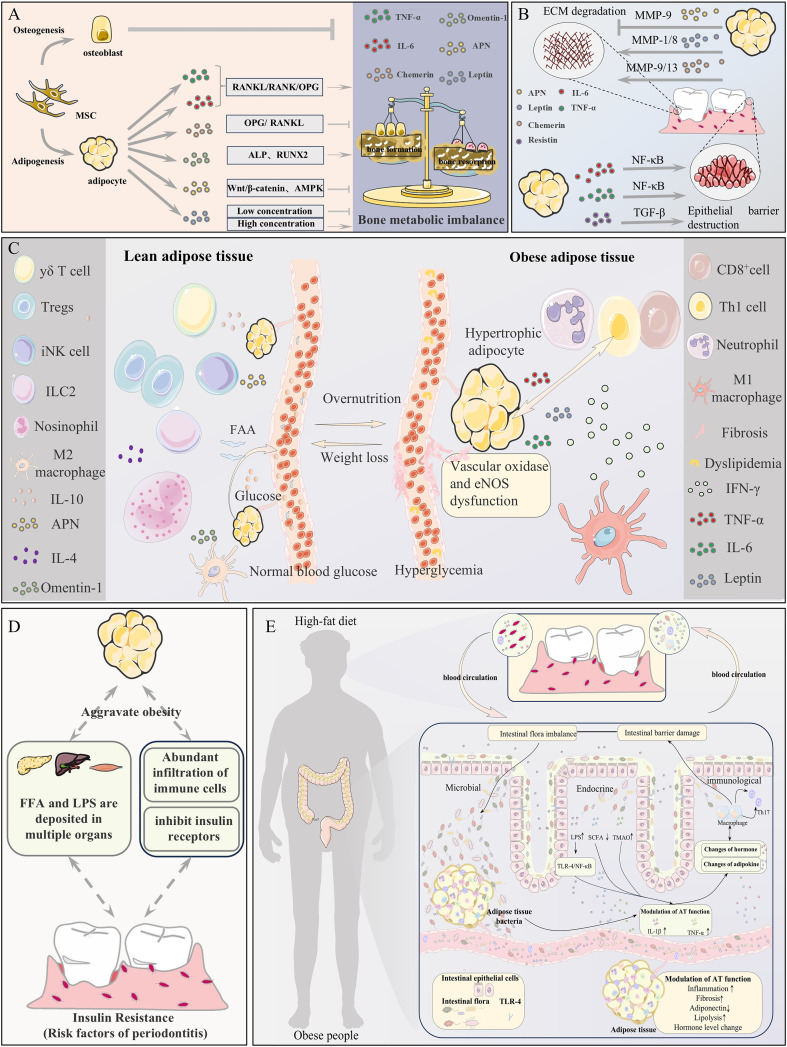
Potential mechanisms linking adipose tissue (AT) to periodontitis. **(A)** AT imbalance disrupts bone metabolism, promoting osteoclast-mediated resorption of periodontal bone. **(B)** AT dysregulation impairs the periodontal extracellular matrix and epithelial barrier function. **(C)** Immune-metabolic imbalance and microcirculatory disturbance in AT collectively exacerbate periodontitis. **(D)** AT dysfunction induces systemic insulin resistance and low-grade inflammation via FFA/LPS, increasing periodontitis susceptibility. **(E)** The Gut-AT-Periodontitis Axis: A high-fat diet induces gut barrier dysfunction and dysbiosis, leading to adipose dysfunction and systemic immune activation, which ultimately accelerates periodontitis via microbial, endocrine, and immune pathways.

Regarding the specific mechanisms of individual adipokines, adiponectin (APN) promotes osteogenic differentiation by activating the Wnt/β-catenin and AMPK signaling pathways while inhibiting osteoclastogenesis. Clinical studies show that APN levels in the gingival crevicular fluid of periodontitis patients positively correlate with osteoprotegerin (OPG) levels (62–64). Furthermore, animal studies confirm that the APN receptor agonist AdipoAI significantly ameliorates alveolar bone destruction in diabetic rats (65, 66). Leptin exhibits a concentration-dependent bidirectional regulatory effect: at low concentrations, it inhibits bone resorption by increasing the OPG/RANKL ratio, while at high concentrations, it promotes receptor activator for nuclear factor-κB ligand(RANKL) expression via the hypothalamus-sympathetic nervous axis (Adrb2 pathway), thereby accelerating bone loss (67–70). Additionally, emerging adipokines like chemerin impair bone formation by suppressing the expression of osteogenic differentiation markers (ALP, RUNX2) in periodontal ligament stem cells (PDLSCs) (71), whereas omentin-1 inhibits osteoclast activity by modulating the OPG/RANKL balance (72). These findings collectively demonstrate the central role of the adipokine network in regulating alveolar bone metabolism (**Figure 2A**).

In summary, AT orchestrates a precisely regulated “Adipose-Bone Axis” governing bone metabolism through two fundamental mechanisms: the inherent fate commitment of multipotent MSCs and its complex adipokine signaling network exemplified by APN, leptin, chemerin, and omentin-1. Dysregulation of this axis manifests as three interconnected pathologies: suppression of osteogenesis by excessive adipogenesis, predominance of pro-inflammatory adipokines, and functional impairment of anti-inflammatory/pro-osteogenic factors. This pathological cascade underpins the link between obesity, chronic inflammatory disorders (epitomized by periodontitis), and alveolar bone destruction. A deeper understanding of these adipokine-mediated regulatory networks—spanning cellular differentiation and signaling pathways—not only provides new insights into impaired bone defect repair in periodontitis but also offers potential therapeutic strategies and molecular targets for intervening in adipose-bone axis imbalance to promote periodontal tissue regeneration (**Table 1**).

**Table 1:** Characteristics of main fat factors and their effects on periodontitis.

Adipokine	Primary source	Relationship with periodontitis	Clinical impact on periodontitis
Adiponectin	WAT	Levels negatively correlated with periodontitis	1. Acts as a macrophage polarization regulator, increasing anti-inflammatory phenotype. 2. Inhibits osteoclast formation stimulated by periodontal pathogens LPS, participates in periodontal bone formation and repair. 3. Promotes the production of cytokines including POSTN, TGF-β, and VEGF to enhance tissue repair.
Leptin	WAT	1. Serum leptin is positively correlated with the severity of periodontitis 2. Local leptin levels in periodontal tissues negatively correlated with the severity of periodontitis	1. Modulates bone metabolism within periodontal tissues through both neural regulation and direct influence on alveolar bone remodeling. 2. Activates peripheral blood B cells, promoting the secretion of IL-6, IL-10, and TNF-α, inducing phosphorylation of JAK2 and STAT3, thereby affecting periodontal immune homeostasis. 3. Promotes ECM degradation in periodontal tissues. 4. Altered oral microbiome composition can impact both periodontal disease and systemic conditions through the leptin signaling pathway.
Chemerin	WAT	Positively correlated with the severity of periodontitis	1. Controls bacterial load and/or composition in the oral cavity. 2. Inhibits ALP and matrix mineralization expression and activity, as well as osteoblast markers and osteogenic differentiation. 3. Activates the inflammatory response in PDLSCs (periodontal ligament stem cells).
Vaspin	Visceral Adipose Tissue	Positively correlated with the severity of periodontitis	1. Enhances the expression of inflammatory cytokines such as TNF-α and IL-6, monocyte chemoattractant protein-1, and plasminogen activator inhibitor-1, increasing the risk of periodontitis.
Omentin-1	Visceral Adipose Tissue	Negatively correlated with the severity of periodontitis	1. Osteoblasts and osteoclasts are direct targets of Omentin-1, playing a crucial role in maintaining normal bone mass. 2. Induces anti-inflammatory factors IL-13 and IL-4, providing protection in systemic inflammation.

#### 4.2.2 Degradation of periodontal ECM and dysfunction of epithelial barrier

In obesity, the dysregulated secretion of adipokines such as leptin, adiponectin, resistin, and chemerin from AT affects periodontal tissues through multiple pathways. On one hand, the secreted adipokines are associated with the degradation and imbalance of ECM. Leptin cooperates with IL-1β to upregulate the expression of MMP-1 and MMP-8 in periodontal fibroblasts; chemerin activates macrophages and fibroblasts via the ChemR23 receptor, promoting the secretion of MMP-9/13 and accelerating collagen degradation (73). Conversely, decreased APN levels weaken its inhibitory effect on MMP-9 while reducing TIMP-1 levels, disrupting the ECM synthesis/degradation balance (74). Moreover, AT modulates epithelial barrier function via the secretion of adipokines. TNF-α and IL-6 diminish the expression of tight junction proteins such as occludin and claudin-1 in gingival epithelial cells through the activation of the NF-κB pathway, thereby enhancing epithelial permeability (75, 76). Resistin induces abnormal keratinization in gingival epithelial cells and promotes epithelial-mesenchymal transition (EMT) through the TGF-β signaling pathway, compromising barrier integrity (77, 78). Barrier disruption facilitates the invasion of bacteria and toxins, activating the TLR pathway and creating a vicious cycle of “inflammation–ECM degradation–exacerbated infection” (79–81). Vaspin protects the ECM by inhibiting Kallikrein 7 protease activity; its elevated levels in periodontitis positively correlate with inflammation severity, suggesting a compensatory repair mechanism (82, 83). The synergistic effect of adipokine-mediated excessive ECM degradation and epithelial barrier dysfunction promotes periodontal pocket formation, attachment loss, and tissue destruction, constituting a critical link in the progression of obesity-associated periodontitis. Targeting the adipokine regulatory network may provide novel strategies for restoring periodontal homeostasis (**Figure 2B**).

#### 4.2.3 Immunometabolic imbalance

The metabolic and immune functions of AT play crucial roles in the development of obesity and periodontitis. Under physiological conditions, white AT primarily serves as an energy reservoir, synthesizing triglycerides from metabolites like blood glucose and free fatty acids (FFA) for storage and mobilizing these reserves when needed. This normal metabolic regulation is essential for maintaining systemic energy balance (2). However, in obesity, adipocytes undergo significant alterations: adipocyte hypertrophy leads to ectopic lipid deposition, while the immune microenvironment of AT undergoes remodeling, characterized by increased infiltration of pro-inflammatory immune cells, including M1 macrophages, CD8^+^ T cells, Th1 cells, and neutrophils, accompanied by fibrosis and aberrant angiogenesis (2). These pathological changes not only disrupt normal AT function but also promote the development of metabolic syndrome through multiple mechanisms.

At the molecular level, abnormal accumulation and activation of macrophages in obese AT are key factors driving insulin resistance. These macrophages secrete large quantities of pro-inflammatory cytokines, including TNF-α and IL-6, which inhibit insulin receptor signaling by activating Ser/Thr kinases while downregulating PPARγ expression and activity, thereby impairing adipocyte differentiation and function (84, 85). This local inflammatory response further propagates systemically, manifesting as elevated circulating pro-inflammatory cytokines and ectopic lipid deposition, ultimately reducing insulin sensitivity in peripheral tissues like skeletal muscle and liver (86). Notably, insulin resistance has been recognized as an independent risk factor for periodontal diseases (87). Chronic hyperglycemia affects periodontal health through multiple pathways, including accumulation of advanced glycation end products (AGEs), alterations in salivary pH, and neutrophil dysfunction. These changes collectively exacerbate the immuno-stress response in periodontal tissues (88). Thus, the immuno-metabolic dysregulation triggered by AT dysfunction constitutes a shared pathological basis for both obesity and periodontitis (**Figure 2C**) (89).

In immune regulation, the role of various adipokines in modulating macrophage polarization is particularly prominent. APN promotes macrophage polarization toward the anti-inflammatory M2 phenotype by inhibiting TNF-α production via the ERK1/2-Egr-1 pathway and enhancing IL-10 secretion through NF-κB signaling (63, 90–92). Conversely, leptin activates the NLRP3 inflammasome to promote M1 macrophage activation, increasing IL-1β and IL-18 release (93). Clinical studies indicate that serum leptin levels in periodontitis patients positively correlate with inflammatory markers like IL-6 and TNF-α (94). Additionally, chemerin mediates macrophage recruitment to inflammatory sites through the ChemR23 receptor and promotes extracellular matrix degradation by upregulating MMP-9/13 expression (73, 80). Vaspin, a serine protease inhibitor, shows significantly elevated levels in obese patients with periodontitis, correlating with changes in inflammatory factors like TNF-α and IL-6. Its levels decrease markedly after periodontal treatment, suggesting its potential as a biomarker for monitoring inflammatory status (82, 83, 95). These findings not only reveal the central role of adipokines in immuno-metabolic regulation but also provide new perspectives for understanding the pathogenesis of obesity-associated periodontitis (**Table 1**).

In summary, the core immuno-metabolic imbalance in obese AT lies in the vicious cycle between immune microenvironment remodeling, particularly macrophage infiltration and polarization dysregulation, triggered by adipocyte hypertrophy and metabolic disorders centered on insulin resistance. Pro-inflammatory immune cells and their cytokines, such as TNF-α and IL-6, impair adipose function and induce systemic insulin resistance; secondary hyperglycemia and metabolic disturbances, such as AGEs accumulation, then exacerbate periodontal immuno-stress and destruction. Key adipokines such as APN, leptin, chemerin, and vaspin regulate macrophage phenotypes and inflammation, and their imbalance not only disrupts adipose homeostasis but also directly serves as molecular bridges for the immunopathological damage in obesity-associated periodontitis. This dysregulated adipose-originated “immuno-metabolic crosstalk” represents the key shared pathological basis between obesity and periodontitis, providing direction for mechanistic exploration, biomarker discovery, and targeted therapeutic development.

#### 4.2.4 Microcirculatory disorders

AT, as a highly dynamic organ, can be classified based on its anatomical location and the cellular composition of different depots (64). Obesity increases the volume of perivascular adipose tissue (PVAT) and induces its dysfunction, manifested by significant alterations in cellular composition and molecular characteristics. PVAT dysfunction involves interactions among multiple aspects, including dysregulation of AT endocrine function, systemic inflammation, vascular dysfunction, and metabolic disturbances. When PVAT dysfunction occurs, the secretion of pro-inflammatory adipokines such as leptin, resistin, TNF-α, IL-6, and IL-1β increases, while the secretion of anti-inflammatory adipokines such as APN and IL-10 decreases. These adipokines can initiate and coordinate the infiltration of inflammatory cells such as T cells, macrophages, dendritic cells, B cells, and NK cells. Protective factors like APN inhibit NADPH oxidase-mediated superoxide generation and enhance nitric oxide (NO) bioavailability in the vascular wall; conversely, inflammatory cytokines such as interferon-γ (IFN-γ) or IL-17 induce aberrant vascular oxidase activity and endothelial nitric oxide synthase (eNOS) dysfunction in endothelial cells, vascular smooth muscle cells, and adventitial fibroblasts. Collectively, these mechanisms link dysfunctional PVAT to vascular dysfunction (96).

Notably, systemic vascular endothelial dysfunction caused by PVAT dysfunction extends to the periodontal microvasculature, leading to reduced local blood flow, inflammatory cell infiltration, ischemia and hypoxia, impaired nutrient/immune cell delivery, and compromised tissue repair capacity (97). Research by Lin et al. suggests that obesity may promote periodontitis by affecting gingival vascular supply and microcirculation (98). Clinical studies comparing gingival biopsy specimens from obese individuals and controls revealed thickened basement membranes in terminal arterioles in the obese group. Furthermore, experimental periodontitis exacerbates inflammation not only by increasing the stromal vascular fraction (SVF) in AT to promote pro-inflammatory cytokine (TNF-α and IL-6) secretion, but also by altering circulating levels of adipokines such as resistin, adiponectin, and leptin (99). Furthermore, PVAT dysfunction is a pivotal factor in insulin resistance and metabolic syndrome linked with obesity. The synergistic impact of elevated pro-inflammatory cytokines (TNF-α, IL-6, and resistin) and reduced APN disrupts insulin signaling pathways, thereby affecting periodontitis (**Figure 2C**) (100, 101).

Regarding microcirculation regulation, different adipokines exhibit distinct effects. Omentin-1 (also known as intelectin-1, ITLN1), primarily expressed in visceral AT, improves endothelial progenitor cell function and promotes vascular repair by inhibiting the p38 MAPK/CREB signaling pathway (72). Leptin, conversely, increases vascular permeability and induces chronic inflammatory responses, exacerbating tissue edema and hypoxia (93). APN promotes reparative angiogenesis in periodontal tissues by stimulating vascular endothelial growth factor (VEGF) secretion (102). Clinical studies indicate that imbalanced angiogenic factors and adipokine levels in the gingival crevicular fluid of periodontitis patients may reflect disrupted periodontal microenvironmental homeostasis (see **Table 1**) (72, 102).

In summary, PVAT dysfunction in obesity is a central hub of immuno-metabolic imbalance: dysregulated secretion of pro-/anti-inflammatory adipokines drives inflammatory infiltration, triggering vascular endothelial dysfunction and insulin resistance. This imbalance directly impairs periodontal microcirculation, causing reduced blood flow, hypoxia-ischemia, impaired vascular repair, exacerbated inflammation, and weakened tissue repair capacity. The regulatory roles of key adipokines in microcirculation—Omentin-1 promoting repair, leptin exacerbating leakage, and APN stimulating angiogenesis—highlight the central impact of adipokine network imbalance on periodontal homeostasis. Elucidating the “immune-metabolic-vascular” tri-network imbalance mechanism mediated by PVAT lays a theoretical foundation for vascular pathology research and targeted therapeutic strategies for obesity-associated periodontitis.

#### 4.2.5 Lipid and glucose metabolism imbalance

AT inflammation impacts periodontal health through multiple mechanisms, with diabetes serving as a crucial intermediary factor. As an established risk factor for periodontitis, the development of diabetes is closely linked to AT inflammation (103). Elevated pro-inflammatory mediators in obesity, including TNF-α, leptin, and resistin, disrupt cellular glucose uptake via suppression of insulin receptor signaling, culminating in hyperglycemia and diabetes (104–106). AGEs produced in this process are pivotal in the link between diabetes and periodontitis, getting worse periodontal hurt through mechanisms including the increase of pro-inflammatory mediator release, induction of abnormal collagen cross-linking, and acceleration of periodontal tissue degradation (106–108).

As a central regulator of systemic energy metabolism, AT dynamically remodels to adapt to nutritional changes. Under physiological conditions, it maintains energy balance by adjusting adipocyte number and volume while coordinating functional changes in stromal and vascular cells (109–112). However, aberrant remodeling induced by obesity triggers adipokine dysregulation, leading to systemic metabolic disorders (31). Animal experiments confirm that the inflammatory response induced by a high-fat diet first appears in AT before spreading to metabolic organs like the liver and muscle (113). This AT-originated inflammation, through various secretory pathways, ultimately causes metabolic damage to multiple organs, including the periodontal tissues (114). Clinical observations demonstrate that dysregulated expression of specific adipokines—including visfatin, APN, and leptin—within periodontal tissues of periodontitis patients correlates significantly with local inflammatory pathology and alveolar bone metabolic dysregulation, revealing a reciprocal pathological circuit that links obesity and periodontitis via metabolic pathways such as lipid/glucose metabolism and oxidative stress (**Figure 2D**) (10).

The essence of this detrimental loop is in the interplay between AT inflammation and lipid metabolism. AT inflammation not only directly causes dyslipidemia, but periodontal infections and their generated inflammatory factors also exacerbate lipid metabolic disorders, forming a positive feedback loop (115). Notably, studies confirm that transplantation of gingiva-derived mesenchymal stem cells not only reduces periodontal inflammation but also improves blood lipid metabolic parameters (77, 78), suggesting periodontal treatment may ameliorate metabolic abnormalities.

At the molecular mechanism level, lipid and glucose metabolism disorders exacerbate periodontal damage through multiple pathways. Obesity-associated insulin resistance promotes AGEs accumulation, disrupting bone collagen structure (72); hyperglycemic environments suppress osteoblast function via oxidative stress. Adiponectin’s role in improving insulin sensitivity through the AMPK pathway provides a novel perspective for comprehending the correlation between metabolic control and periodontal health (refer to **Table 1**) (102). Clinical studies further confirm that the severity of periodontitis in type 2 diabetes patients significantly correlates with the serum leptin/APN ratio, highlighting the critical role of adipokine balance in periodontitis development (92, 94).

In summary, AT inflammation is the key initiating factor driving lipid metabolism disorders, specifically dyslipidemia, and glucose metabolism imbalance, including insulin resistance, hyperglycemia, and AGEs accumulation. This metabolic dysregulation state, particularly diabetes as the core intermediary, significantly exacerbates periodontal inflammation and bone resorption through various molecular pathways, such as AGEs-mediated tissue destruction, oxidative stress inhibiting bone formation, and adipokine imbalance. Concurrently, local periodontal infection and inflammation exacerbate systemic and AT metabolic dysregulation through positive feedback mechanisms, establishing a self-perpetuating pathological cascade. This cycle initiates with AT inflammation, propagates metabolic imbalance, induces periodontal destruction, and ultimately intensifies metabolic dysregulation. Its core pathological driver involves direct and indirect toxicity from systemic metabolic disorders—mediated by aberrant adipose remodeling—targeting periodontal tissues. Crucially, the adipokine regulatory axis, exemplified by leptin-to-APN ratios, functions as a central control node. Periodontal interventions such as stem cell transplantation demonstrate potential to improve metabolic parameters, revealing not only the intrinsic linkage in metabolic periodontitis but also providing foundational insights for future adipo-metabolic axis-targeted therapies to disrupt this cycle and restore periodontal health.

#### 4.2.6 Periodontitis under the crosstalk between the Gut-AT axis

The study of gut microbiota-AT interactions is a relatively new field, with gut microbiota recognized as crucial regulators of host metabolic homeostasis and energy balance (116). Lam et al. demonstrated that long-term feeding with high saturated fats leads to gut inflammation and barrier dysfunction, associated with specific changes in the gut microbiome (117); meanwhile, mesenteric fat adjacent to the gut, rather than more distant depots, exhibits pronounced pro-inflammatory properties, indicating a connection between the gut and visceral fat inflammation and systemic metabolic dysfunction (118). From this, it can be inferred that obesity and overfeeding affect the gut microbiome and gut permeability, leading to chronic low-grade inflammation in AT. Additionally, researchers have revealed that the gut microbiota can induce biological changes in AT through secreted molecules that enter systemic circulation, reaching and acting on adipocytes and other cells within fat depots. These secreted molecules can be broadly categorized into microbial metabolites and microbial cell components. Metabolites produced by the microbiota, such as short-chain fatty acids, secondary bile acids, and bioactive lipids derived from the endocannabinoid system, enhance BAT activity and WAT browning, playing significant roles in the development of obesity and related metabolic diseases. Currently, natural compounds such as polyphenols, terpenoids, alkaloids, and prebiotics are potential browning agents in white AT, which can alleviate systemic metabolic diseases by modulating gut microbial metabolites (119–123). Conversely, common microbial components, such as translocated Gram-negative gut bacteria or increased LPS, can lead to increased gut permeability, also known as gut barrier dysfunction, inducing metabolic endotoxemia (124). Gut-derived bacteria or elevated LPS colonize AT, and the TLR activation mediated by endotoxins will ultimately lead to inflammation and overall dysfunction of AT.

The close relationship between AT and the gut microbiota has significant implications for periodontitis. It is well known that BAT is crucial for thermogenic energy expenditure, and the reduced fatty acid oxidation capacity of BAT in obese individuals is a key factor in the further progression of obesity (118). Emerging research establishes that gut microbiota-mediated regulation of WAT browning and BAT activity critically sustains adipose functionality. The microbial metabolite trimethylamine-N-oxide (TMAO) is increasingly implicated in cardiovascular pathogenesis and inflammatory responses (125). TMAO biosynthesis originates from gut microbial metabolism of dietary choline and L-carnitine, which generate trimethylamine (TMA). Hepatic flavin monooxygenase 3 (FMO3) subsequently oxidizes TMA to yield TMAO (126, 127). Clinically significant TMAO elevation correlates with type 2 diabetes progression and obesity-related metabolic alterations (128). Schugar et al. proposed that the TMA/FMO3/TMAO pathway acts as a microbiota-to-host endocrine axis mediating gut-AT crosstalk, and deleting Fmo3, which produces TMAO, or reducing TMAO precursor TMA would increase gonadal WAT browning and prevent obesity in mice (129). Since TMA is derived from a high-fat diet and entirely produced by the gut microbiota, dietary interventions or targeting specific microbes that produce TMAO may have therapeutic implications for obesity and its complications. Interestingly, clinical studies show elevated circulating TMAO levels in periodontitis patients, suggesting TMAO might be a new clue to explore the relationship between periodontal infection and the gut-AT axis. TMAO has also been shown to be abnormally increased in osteoporosis, promoting osteoclast differentiation and inducing bone loss in mice through activating ROS-dependent NF-κB signaling pathways, impacting periodontal health (130). Furthermore, gut microbiota metabolites such as short-chain fatty acids (SCFAs) and secondary bile acids are associated with the development of oral inflammation (122, 131). These studies indicate that molecules secreted by AT under the influence of the microbiota affect periodontitis.

Obesity often impairs the periodontal epithelial barrier function, allowing periodontal pathogens and toxic products to invade deep tissues and systemic circulation through compromised periodontal pockets, activating local inflammatory responses and directly affecting the periodontal microenvironment. Maciel et al. found that obese patients with periodontitis had higher quantities of periodontal pathogens compared to normal-weight periodontitis patients, and the proportion of suspected periodontal pathogen Fusobacterium in subgingival biofilms increased with BMI, leading to altered host immune responses to plaque-derived antigens and thus inducing periodontitis (132). Additionally, periodontitis, a chronic inflammatory disease caused by complex interactions between subgingival biofilms and host immune-inflammatory responses, results in oral microbiota dysbiosis, which affects various systemic diseases and indirectly impacts the periodontal microenvironment (133). Studies have shown that orally administered periodontal pathogen Porphyromonas gingivalis can enter the gut through the digestive tract, causing gut microbiota dysbiosis and systemic inflammation, downregulating genes that improve insulin sensitivity in AT (C1qtnf9, Irs1, and Sirt1), and upregulating genes related to lipid droplet formation and gluconeogenesis (Plin2, Acox, and G6pc), inducing insulin resistance and increasing susceptibility to periodontitis. Animal experiments demonstrate that fecal microbiota transplantation from obese mice to periodontitis mice significantly increases alveolar bone resorption (13). According to a clinical study by Laugerette et al., overfeeding increases postprandial endotoxemia (134). AT inflammation promotes the secretion of dozens of bioactive molecules by adipocytes, such as leptin, resistin, tumor necrosis factor-α (TNF-α), interleukins (IL-1, IL-6, IL-8, and IL-10), growth factors, complement components, angiotensinogen, plasma fibrinogen, activator-1 (PAI-1), and many other substances. These bioactive molecules trigger endotoxemia-related systemic inflammation, potentially exacerbating periodontal inflammation. These studies collectively indicate that the crosstalk between the gut-AT axis has direct and indirect effects on periodontitis, presenting a broad research prospect (**Figure 2E**).

## 5 AT: an inflammatory organ that can not be ignored in periodontal treatment

### 5.1 Adipokines: the common mediator linking periodontitis and systemic inflammation

Chronic inflammation is widely recognized as a common link between periodontitis and inflammatory diseases such as obesity and type 2 diabetes mellitus (T2DM). Researchers suggest that pro-inflammatory cytokines and bacterial products are secreted from infected gingiva into the circulation, potentially affecting systemic health. However, recent studies have shown that AT, especially visceral fat, is a major site for the production of inflammatory molecules, including C-reactive protein (CRP), IL-6, and TNF-α. AT inflammation is a significant cause of systemic chronic inflammation. These can contribute to oxidative stress and act as pro-inflammatory or anti-inflammatory mediators in periodontal inflammation (135, 136). These adipokines play crucial roles in metabolism and inflammation, and their dysregulation is key to inflammation and immune responses, bone and fat metabolism, energy expenditure, and insulin sensitivity regulation, potentially inducing the development of periodontitis.

On the other hand, recent clinical studies have found that periodontitis affects the circulating levels of adipokines, including resistin, adiponectin, and leptin, promoting inflammation. Given this, exploring AT inflammatory responses induced by periodontitis seems increasingly important, as this could reveal the interrelationship between periodontitis and systemic inflammatory states. Targeting adipokines might emerge as a promising therapeutic strategy for periodontitis.

### 5.2 Adipose stem cells: the potential star of periodontal tissue regeneration

AT is a source of multipotent mesenchymal progenitor cells capable of differentiating into various lineages, including adipogenesis, osteogenesis, and chondrogenesis, which is linked to bone metabolism. MSC-based therapies have become a promising approach for periodontal tissue regeneration (137). Preclinical studies have shown that the delivery of MSCs—including dental MSCs such as PDLSCs, dental pulp stem cells (DPSCs), and dental follicle stem cells (DFSCs), as well as non-dental MSCs—produces reliable and effective therapeutic results in periodontitis models. Compared to other MSCs, adipose-derived stromal/stem cells (ASCs) are considered promising candidates for MSC-based therapies due to their easy acquisition, abundance, and strong immunomodulatory capacity. Pre-adipocytes, mature adipocytes, vascular smooth muscle cells, endothelial cells, fibroblasts, and resident monocytes/macrophages from subcutaneous fat are essential in periodontal regeneration. Furthermore, ASCs are abundant in AT and maintain their stemness after several passages, whereas the number of PDLSCs is inherently limited.

Early studies have confirmed that the transplantation of ASCs with biomaterials or platelet-rich plasma in alveolar bone defects promotes periodontal tissue regeneration (138, 139). The combination of AT with fibrin sealants demonstrates potential in promoting the regeneration of periodontal membrane tissue. Notably, adipose-derived multipotent progenitor cells (ADMPCs) have emerged as a widely investigated viable cell source for regenerative medicine applications. ADMPCs share key characteristics with other mesenchymal stem cells and offer distinct advantages, including a relatively straightforward harvesting procedure and minimal donor site morbidity. Current research indicates that the periodontal microenvironment may promote the growth and differentiation of ADMPCs into periodontal tissues, positioning them as a promising therapeutic approach for restoring functional periodontal structures (140).

### 5.3 AT-immune axis: a double-edged sword for periodontitis treatment

AT, as an important endocrine and immunoregulatory organ, is like an “immune double-edged sword” hidden in the body. It plays a key role in maintaining systemic immune homeostasis by secreting adiponectin, leptin, and other fat factors, which provides a new idea for the treatment of periodontitis. However, the other side of this “double-edged sword” is equally sharp: under pathological conditions such as obesity, it will quickly transform into a pro-inflammatory phenotype, and a large amount of inflammatory factors will aggravate periodontal damage.

To guide this “double-edged sword” to treatment accurately, we still face multiple challenges: First, how to control its two-way regulation characteristics and prevent it from “defecting” as an accomplice to inflammation is the core problem (141). Secondly, it is still unknown whether adipose-derived stem cells, as the key to repair, can “survive,” “home” efficiently, and work stably in the high inflammatory environment of periodontitis, which puts forward higher requirements for local delivery technology (142, 143). Thirdly, the network of adipokines is complicated, and the intervention of a single target is like a drop in the bucket, which makes it difficult to reverse the overall immune imbalance. Finally, the huge heterogeneity of AT among individuals makes the treatment response difficult to predict, calling for the establishment of personalized programs (139).

### 5.4 The gut-adipose-periodontal axis: a new perspective of periodontitis regulation

Beyond direct immune functions within AT, recent research also highlights interactions with the gut microbiome. According to recent research, Imeglimin, a potential drug that promotes energy expenditure and gut integrity, can improve overall metabolism by targeting BAT and gut microbiota in obese model mice (144). Imeglimin administration results in significant changes in the gut microbiota, including an increase in the genus *Akkermansia*, and alleviates obesity-associated gut pathology. Furthermore, clinical studies have shown that gut probiotics such as *Akkermansia* can inhibit periodontitis by inducing adaptive immune responses in the gut that regulate host immune responses (145, 146). These findings imply that AT may indirectly affect periodontitis through a gut–adipose axis, underscoring the therapeutic potential of targeting immune pathways in AT.

Although the concept of a gut–adipose–periodontal axis offers an integrative framework for understanding the links between periodontitis and systemic conditions such as diabetes and obesity, clinical research in this area remains limited. Currently, most supporting evidence comes from animal studies or cross-sectional human analyses. While animal models cannot fully replicate the complex physiological and immune milieu of humans, observational human studies can only reveal correlations rather than establish causality (147). A major limitation is the scarcity of large-scale prospective cohort studies and interventional trials that directly test whether modulating gut microbiota or adipokine networks can improve periodontal health. Additionally, substantial interindividual variation in gut microbiome composition, dietary habits, and genetic background introduces important confounding factors, complicating the reproducibility of findings (148, 149). Other constraints—such as small sample sizes, short follow-up periods, and the lack of standardized assays for microbiome and inflammatory biomarkers—further undermine the reliability and generalizability of current conclusions (6, 150). Therefore, future research should prioritize well-designed, long-term intervention studies with rigorous follow-up to bridge the gap between mechanistic insight and clinical application, ultimately informing novel strategies for prevention and treatment along this axis.

## 6 Future prospects

Recognizing AT as a central inflammatory endocrine organ in obesity-related periodontal disease necessitates integrating obesity management, with an emphasis on weight loss and metabolic improvement, directly into periodontal care. Clinicians should adopt interdisciplinary collaboration: periodontal specialists must assess metabolic parameters like BMI, waist circumference, and blood glucose for risk stratification and treatment planning, while other healthcare providers evaluate periodontal health in obese patients. Critically, obesity interventions intrinsically support periodontal therapy; lifestyle modifications that reduce weight lower systemic inflammation and improve clinical outcomes such as probing depth and attachment loss. Inflammatory mediators from AT, such as leptin, adiponectin, TNF-α, and IL-6, offer promising biomarkers for risk stratification, disease monitoring, and precision therapies targeting adipokine pathways or host modulation. Future efforts should prioritize developing integrated obesity-periodontal interventions, validating adipokine-based biomarker panels, and elucidating mechanistic adipose-periodontal crosstalk. Public health initiatives must unify oral and systemic health promotion to optimize resources and enable comprehensive, precision-based care.

## 7 Conclusion

The global obesity epidemic demonstrates that AT functions beyond a passive energy reservoir as a dynamic endocrine-immune interface. Chronic low-grade inflammation stemming from immune dysregulation, characterized by M1 macrophage polarization and Th17/Treg imbalance, coupled with aberrant secretion of inflammatory adipokines including TNF-α, IL-6, and leptin, induces systemic immunometabolic perturbations. These disturbances directly compromise bone remodeling, microvascular integrity, and immune surveillance, thereby increasing periodontal tissue susceptibility to microbial invasion and impairing regenerative capacity. Critically, adipose-driven inflammation and periodontitis sustain a bidirectional pathogenic cycle: obesity aggravates periodontal destruction, while periodontal infections potentiate systemic inflammation and accelerate metabolic comorbidities. Interrupting this cycle necessitates integrated interventions in which combining obesity management through weight loss and anti-inflammatory strategies with periodontal therapy synergistically enhances clinical outcomes, evidenced by reduced probing depths, attenuated systemic inflammation, and restored metabolic equilibrium. Future research priorities encompass: developing precision therapeutics targeting adipose-specific inflammatory pathways; decoding mechanistic foundations of adipose-periodontal immune crosstalk; validating adipose-centric treatments in obese periodontitis cohorts; and establishing inflammatory adipokines as early risk-stratification biomarkers. Reconceptualizing AT as a central inflammatory nexus reframes the obesity-periodontitis synergy, demanding multidisciplinary collaboration to alleviate the dual burden of metabolic and oral diseases.
